# Insight into the impact of diabetes mellitus on the increased risk of hepatocellular carcinoma: mini-review

**DOI:** 10.1186/2251-6581-13-57

**Published:** 2014-05-22

**Authors:** Maisa Mahmoud Ali Kamkar, Rasheed Ahmad, Osama Alsmadi, Kazem Behbehani

**Affiliations:** 1 Dasman Genome Center, Biomedical Research Department, Dasman Diabetes Institute, Dasman, Kuwait; 2 Immunology and Innovative Cell Therapy Unit, Biomedical Research Department, Dasman Diabetes Institute, Dasman, Kuwait; 3 Dasman Diabetes Institute, Dasman, Kuwait; 4 Department of Biomedical Research, Genetics and Genomics Unit/Dasman Genome Center, Dasman Diabetes Institute, P.O.Box 1180, Dasman 15462, Kuwait

**Keywords:** Apoptosis and inflammation, Diabetes mellitus, DNA methylation or histone modifications, Epigenomic reprogramming, Hepatocarcinogenesis, Hepatocellular carcinoma

## Abstract

Hepatocellular carcinoma is a multifactorial disease which is associated with a background of many causal risk factors. Diabetes mellitus however is one of the most common co-morbid illnesses found in hepatocellular carcinoma patients that are significantly associated with worsening of hepatocellular carcinoma development, patient prognosis and survival. Therefore, efforts have been focused on understanding the mechanisms underlying progression of hepatocellular carcinoma onset and development especially in diabetic patients. To our knowledge, there are no reports which address the impact of tumor necrosis factor alpha (TNF-α) and interleukin-6 (IL-6) along with epigenetic regulations associated with increased risk of hepatocellular carcinoma confounded by diabetes mellitus. Therefore, this mini-review focuses on the possible intermediary mechanisms involved in worsening the onset and progression of hepatocellular carcinoma development confounded by diabetes mellitus. The first approach is to look at the role of inflammatory mediators (TNF-α and IL-6) in apoptosis and inflammation during hepatocarcinogenesis through monitoring levels of apoptotic regulators, B-cell lymphoma 2 protein which is encoded by BCL2 gene and apoptosis regulator BAX known as bcl-2-like protein 4 which is encoded by the BAX gene. The second approach is to focus on the possible epigenomic reprogramming that drives hepatocellular transformation since epigenetic modification of DNA is a key feature in the pathogenesis of hepatocarcinogenesis. Both approaches may suggest role of using Bcl2 and Bax as apoptotic and inflammatory markers for hepatocellular carcinoma detection as well as the importance impact of DNA methylation, hypomethylation or histone modifications as attractive candidates for early-detection biomarkers of hepatocellular carcinoma.

## The association between diabetes mellitus and the risk of hepatocellular carcinoma

Hepatocellular carcinoma (HCC) is the fifth most common malignancy in the world with high morbidity and mortality but its pathogenesis remains unclear [1]. Because primary liver cancer is a growing concern showing poor prognosis due to its rapid infiltrating power and complicating liver cirrhosis; more attention were given to address the causal risk factors that could be preventable and/or treatable [2]. Several risk factors have been identified that contribute to the international burden of HCC such as chronic infection with hepatitis B virus (HBV) and hepatitis C virus (HCV), alcoholic liver disease, non-alcoholic steatohepatitis (NASH), diabetes mellitus (DM), obesity, intake of aflatoxins-contaminated food, tobacco smoking, excessive alcohol drinking and genetically inherited disorders (hemochromatosis, α-1 anti-trypsin deficiency, porphyrias); [3]. HCC is phenotypically and genetically heterogeneous tumor, reflecting in part the heterogeneity of etiologic factors involved in (i) the onset of HCC development that is also influenced by age, gender and ethnic differences, (ii) the complexity of hepatocyte functions and epigenome leading to neoplastic transformation and (iii) the late stage of HCC development [2].

Because the liver plays a crucial role in glucose metabolism, it is not surprising that DM is an epiphenomenon of many chronic liver diseases such as chronic hepatitis, fatty liver, liver failure and cirrhosis [4]. DM is a metabolic disorder characterized by hyperglycemia which may predispose the liver to relative insulin resistant due to inadequate secretion or receptor insensitivity to endogenous insulin. DM is one of the leading causes of blindness and the most common cause of end-stage renal disease and cardiovascular complications in developed countries. In recent years, DM has been associated with increase risk for several malignancies including breast, colon, kidney, liver, endometrium and pancreatic cancers [5]. In addition, DM as part of the insulin resistance syndrome, has been implicated as a risk factor for non-alcoholic fatty liver disease (NAFLD), including its most severe form non-alcoholic steatohepatitis (NASH); which has been identified as a cause of both cryptogenic cirrhosis and HCC. DM has been reported to cause a 2.5-fold greater risk of HCC, however this significant association was independent to hepatitis viral infections (HBV and/or HCV) or alcohol consumption as examined from 10 previously reported studies; those reports account for ~90% of HCC cases [1,6]. Many case reports and case reviews of HCC in NASH have showed the association of DM and obesity on the increased risk of HCC and have implicated age and advanced fibrosis as significant risks. Insulin resistance and the resulting inflammatory cascade, which are associated with the development of NASH appears to mediate hepatocarcinogenesis in HCC [1].

The association between DM and HCC has been demonstrated in both case–control and cohort studies, suggesting that DM is independent risk factor for the development of HCC. These several lines of evidence includes; (i) findings that insulin resistance and DM were shown to increase the progression of liver diseases that preceded the development of HCC [6,7]; (ii) findings showing that 2.5 fold increase in the risk of HCC was reported in patients with DM [8,9] attributed with the prolonged DM disease duration [10-12]; (iii) findings demonstrating the synergistic interaction between DM and other HCC risk factors [11,13,14]; (iv) findings showed significant association of DM for the recurrence of HCC after treatment [15,16]; (v) findings suggesting significant biological plausibility underlying the association between DM and HCC [7,17].

Nevertheless, the exact pathophysiological mechanisms of this significant association are still unclear. In this mini-review, we aim to further analyze this relationship by evaluating the role of possible intermediary mechanisms that could be associated with the onset and progression of HCC development in the presence of DM. We will mainly focus on; (i) pro-inflammatory gene products (TNF-α and IL-6) and (ii) the epigenetic pathogenesis of DM on the development of HCC.

## Possible intermediary mechanisms involved in worsening the onset and progression of hepatocellular carcinoma confounded by diabetes mellitus

### TNF-α/ NF-kβ and IL-6/STAT-3 signaling pathways effect on hepatocellular carcinoma development

Malignant transformation of hepatocytes may occur through a pathway of increased liver cell turnover induced by chronic liver injury and regeneration in the context of inflammation, immune response and oxidative DNA damage [18]. This is evidence to suggest that chronic inflammation in the individuals with diabetes mellitus type 2 (T2DM) may influence certain cancers via cytokines. Cancerous and precancerous tissue show signs of inflammation, which is caused by the infiltration of the immune cells into the tissues. The presence of inflammatory cytokines is important constituents of the local environment of tumors in certain type of cancer including HCC. These cytokines cause oncogenic changes that promote tumor development by blocking apoptosis and increasing the survival of malignant cells [19]. Although an elevation in the circulating levels of tumor necrosis factor-alpha (TNF-α), interleukin-6 (IL-6), interleukin-8 (IL-8), interleukin-12 (IL-12), interleukin-1-beta (IL-1β) and transforming growth factor beta (TGF-β-1) has been observed in obese and diabetic individuals [20-24]; plasma levels of interleukin-10 (IL-10) were found elevated in obese individuals thus, decreased in diabetics [25].

Cytokines such as IL-1β, TNF-α, IL-8 and IL-6 have been involved in chronic liver inflammation and pathogenesis of various liver diseases; among which IL-6 and TNF-α, are thought to be most important [26-28]. In this regard, TNF-α and IL-6 were found to positively correlated with the progression of HCC development in humans [29-31].

IL-6 is a pleiotropic cytokine secreted by T cells, macrophages, kupffer cells, and adipose tissue. Similarly, TNF-α is secreted by several inflammatory cell types, including monocyte/macrophages, neutrophils, T-cells, kupffer cells and adipose tissue [26,32]. Increased circulating levels of IL-6 and TNF-α have been found in animal models and patients with liver disease including HCC [33]. Moreover, studies have revealed that the levels of TNF-α and IL-6 are significantly increased in rats bearing hepatoma, which reflects an aggressive inflammatory response correlated with tumor induction [34]. In obesity and DM, tremendous amount of the tumor-promoting cytokines IL-6 and TNF-α are produced, which is thought to promote HCC development [35,31]. Moreover, DM-mediated inflammatory responses lead to the production of cytokines, which act as growth and angiogenic factors for transformed cells. DM has confounding effects on the factors of inflammation, proliferation and anti-apoptosis in HCC, which explains why HCC and DM co-occur twice as frequently as expected. Furthermore, it has been reported that diabetic patients with HCC are suffer more frequently with liver injury and tumor growth as compared to non-diabetics [19].

**Figure 1:**
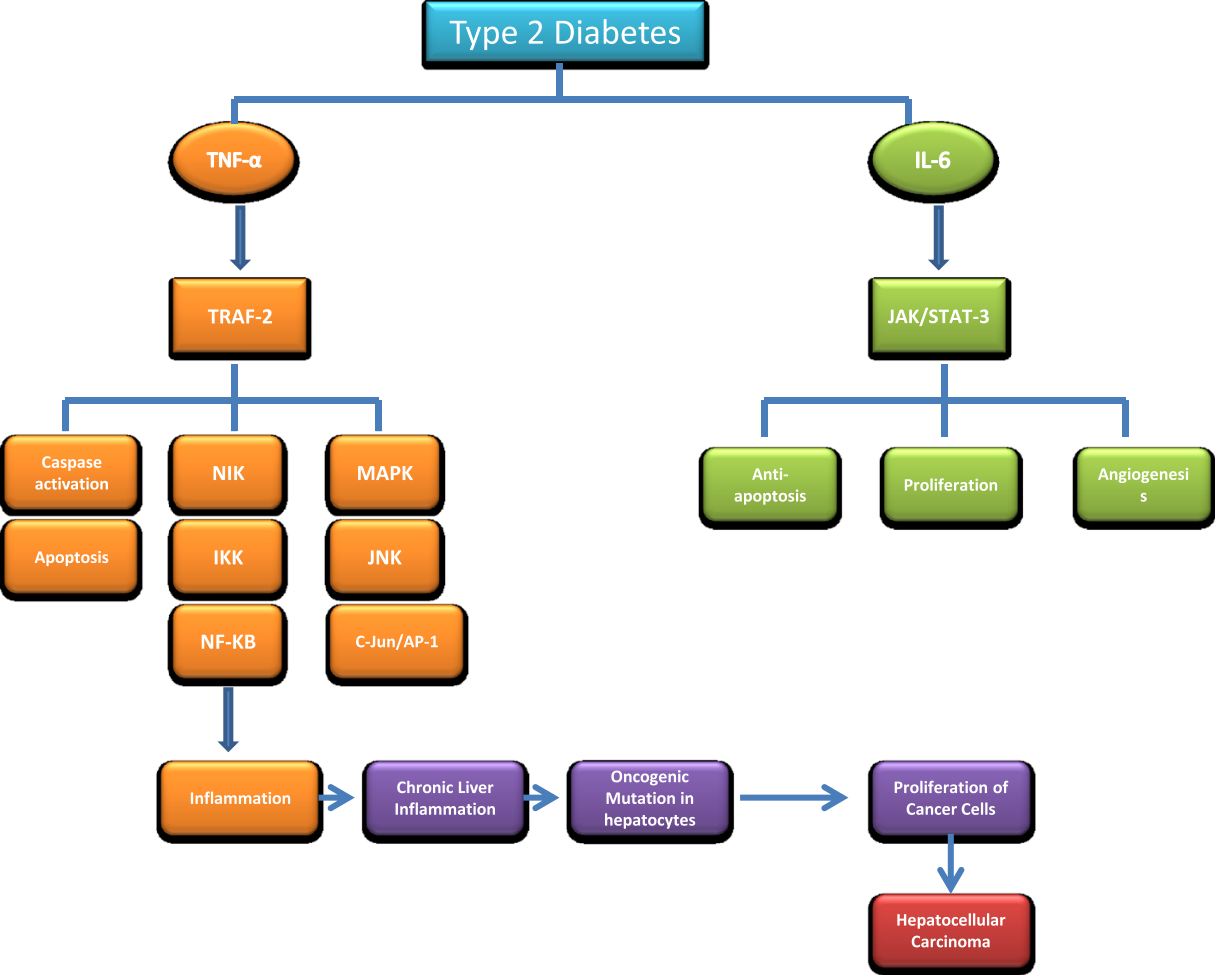
**Oncogenic impact of IL-6/STAT-3 and TNF-α/NF-kβ signaling pathways on the development and progression of HCC.** Obesity induced type-2 diabetes, produces IL-6 and TNF-α by accessory cells, adipose tissue and Kupffer cells. These inflammatory markers (IL-6 and TNF-α) activate the downstream signaling molecules STAT-3 and NF-kβ in residual liver cells, respectively. This activation of NF-kβ and STAT-3 signaling pathways are likely to be involved in critical processes such as anti-apoptosis, proliferation and angiogenesis, which contribute to hepatocarcinogenesis.

IL-6 and TNF-α are involved in multiple signalling pathways, which ultimately lead to liver injury, inflammation and HCC development (Figure 1). Chronic inflammation in diabetic patients may promote carcinogenesis through multifaceted processes such as activation of signal transducer and activator of transcription 3 (STAT-3) and nuclear factor kappa-light-chain-enhancer of activated B cells (NF-kβ) transcription factors following the production of tumor growth factors or induction of angiogenesis. It is known that STAT-3, activated by IL-6 in hypatocytes, promotes HCC cell growth *in vitro* and *in vivo *[36]. In addition, activated IL-6/STAT-3 pathways have been observed in liver cancer and are thought to be important factors in the initiation, development, and progression of HCC [37,38]. Furthermore, blockade of STAT-3 may have therapeutic potential in preventing and treating liver cancer [39]. Since serum concentrations of IL-6 are elevated in diabetic patients, it is reasonable to surmise that IL-6-activated STAT-3 signaling pathway plays play a role in HCC development in DM. The oncogenic role of constitutively activated STAT-3 is driven through the up-regulation of cell survival proteins (Bcl-xL, BCL-2) and cell cycle regulators (c-Myc, cyclin D) [38,40-42]. The second main HCC oncogenic pathway is TNF-α-mediated activation of NF-kβ, which is a key regulator of inflammation that provides a mechanistic link between inflammation and apoptosis during carcinogenesis [43]. Constitutively increased NF-kβ activation has been observed in tumor tissues [44]; however, IKK-α, a critical kinase for NF-kβ activation, is necessary to produce malignant properties in liver cancer [45]. NF-kβ activation not only protects tumor cells against cell death but also provides essential growth factors to non-parenchymal cells of the liver, such as Kupffer cells [46,47]. TNF-α-mediated NF-kβ activation has both positive and negative impact on our immune system [48,49]. An imbalance in TNF-α mediated NF-kβ could lead to several types of inflammatory disorders. In DM, a balance shift towards a prolonged activation of IKK-α/NF-kβ, and simultaneous blockade of apoptosis, is essential for the inhibition of tumor growth. TNF-α induced activation of mitogen-activated protein kinases (MAPKs) are also significant factors for tumour growth. TNF-α levels were shown to be higher in obese and diabetic individuals and to correlate with insulin resistance and liver disease [27,32,50]. In addition, increased STAT-3 and NF-kβ were shown to be correlated with impaired insulin sensitivity and more advanced development of T2DM. Furthermore, systemic abnormalities in the activation of STAT-3 and NF-kβ in subjects with DM provide clinically important milieu for increased risk of severe HCC development [51,52]. Since the presence of TNF-α and IL-6 is considered to be a possible risk factor for the development of HCC in diabetic individuals, DM is thought to be a major risk factor for the development of aggressive HCC. In conclusion, these studies suggest that the presence of liver inflammation in the context of DM, leads to the exposure of hepatocytes to increased levels of IL-6 and TNF-α, which promote the activation of JAK/STAT-3 and IKKα/NF-kβ signaling pathways, followed by lack of apoptosis, and consequently uncontrolled proliferation of hepatocytes; this results in initiation and promotion of HCC development [53].

### Epigenomic reprogramming that drives hepatocellular transformation

Multifactorial diseases such as DM and HCC are far more complex compared to single gene disorders; whereby multiple genes in addition to non-genetic components dictate the pathogenic manifestations of the disease. Further sophistication layer is added onto these disorders from modifications on the genetic material in cells and tissues. These are referred to as “epigenetics factors”. Epigenetic (meaning “above” genetics) inheritance is essential for the development of critical cellular processes such as gene transcription, differentiation and protection against viral genomes. Aberrant epigenetic states may predispose to genetic changes or vice versa [54-56]. Therefore, epigenetic and genetic mechanisms may work together to silence key cellular genes and destabilize the genome, leading to oncogenic transformation and observed complexity and heterogeneity in human cancers. The main epigenetic modifications that can alter gene expression and tissue-specific cellular changes [57] are DNA methylations, histone modifications, and non-coding RNAs (micro and long non-coding RNAs); that together impact on transcriptional regulation of wide range of genes [58,59]. Thus, unregulated epigenetic events play key role in pathogenic mechanisms affecting the expression of many genes including tumor-suppressor genes and cancer-associated genes as has been observed in broad human cancers [60]. Moreover, a number of studies suggested that a frequent loss of heterozygosity (LOH) in chromosome 8p in HCC cases which leads to inactivation of the Deleted in Liver Cancer 1 gene (DLC-1) may play a pivotal role in HCC development [61]. Thus, in the late stage of HCC development; somatic mutations in several tumor suppressor genes (such as TP53, p16 and RB); oncogenes (such as c-MYC and β-catenin) and other cancer-associated genes including E-cadherin and cyclin D1 have also been observed. However, the significance and sequence of these genetic events remain to be established [61,62].

Recent data suggest that the epigenetic pattern is age-dependent influencing key mitochondrial respiratory chain genes [63,64]. An example is the complex 4 COX7A1 protein (which is a target of age-related DNA methylation) as evidenced from its expression reduction in muscle tissue from diabetic patients [64,65]. In young and elderly twin studies; COX7A1 gene promoter methylation was shown to be increased in skeletal muscles of elderly compared with young twins, and that was reciprocal to expression pattern of COX7A1 gene. The transcript level of COX7A1 gene in skeletal muscle was associated with increased *in vivo* glucose uptake [64]. Polymorphism can also lead to generation of DNA methylation sites (CpG dinucleotides). In addition, putative transcription factor binding site in the NDUFB6 promoter are associated with increased DNA methylation, decreased gene expression, and decreased *in vivo* metabolism with increasing age [66]. The impact of aging on T2DM is also evident from data on the hepatic function of glucokinase enzyme glucose utilization, where its activity is decreased in the diabetic patients’ liver [67]. Further evidence for epigenetic role was demonstrated in a recent study in which significant differential DNA methylation profiling was indicated when comparing pancreatic islets from type 2 diabetics with non-diabetic controls; where 276 CpG loci associated with 254 genes were uncovered. These methylation signatures were absent in peripheral blood cells of diabetic individuals, and couldn’t be experimentally induced in non-diabetic islets by exposure to high glucose, suggesting tissue-specific methylation patterns [68]. There is also the potential role for epigenetic control microRNAs (miRNAs) on chromatin-modifying enzymes leading to effects in gene expression. Histone modifications and changes in chromatin structure can affect transcription and expression of miRNAs [69]; which are associated with the regulation of pathogenic pathways critical in insulin secretion, cholesterol synthesis and fat metabolism [70-72]. Increased telomeric activity and hTERT expression in HCC cases were also reported. In one study on 106 hepatic tissues with and without HCC; hTERT expression has correlated inversely with DNA methylation levels in normal tissues compared to tumor tissues in a mechanism thought to be regulated by DNA methylation and histone H3-K9 modifications [73].

Furthermore, genome-wide oncogenes promoter demethylation was detected during HCC progression [74]. This demethylation was accompanied by selective regional hypermethylation in the CpG islands leading to silencing of antitumor genes like tumor suppressor genes, proliferation inhibitor genes, in addition to apoptotic and DNA repair genes [75,76]. A proposed mechanism for HCC is suggested to occur via activating inflammatory response components’ (NF-κB and JAK/STAT) to induce epigenetic changes resulting in switching on the long-term oncogenic memory system in hepatocytes [77]. The epigenetic switch in turn would contribute to a chronic inflammatory response during the course of altered gene expression concordant with a positive feedback loop to aggravate a chronic state of inflammation. These uncontrolled epigenetic-driven transcriptional alterations ultimately promote hepatocytes proliferative and oncogenic transformations.

## Conclusions

**Figure 2:**
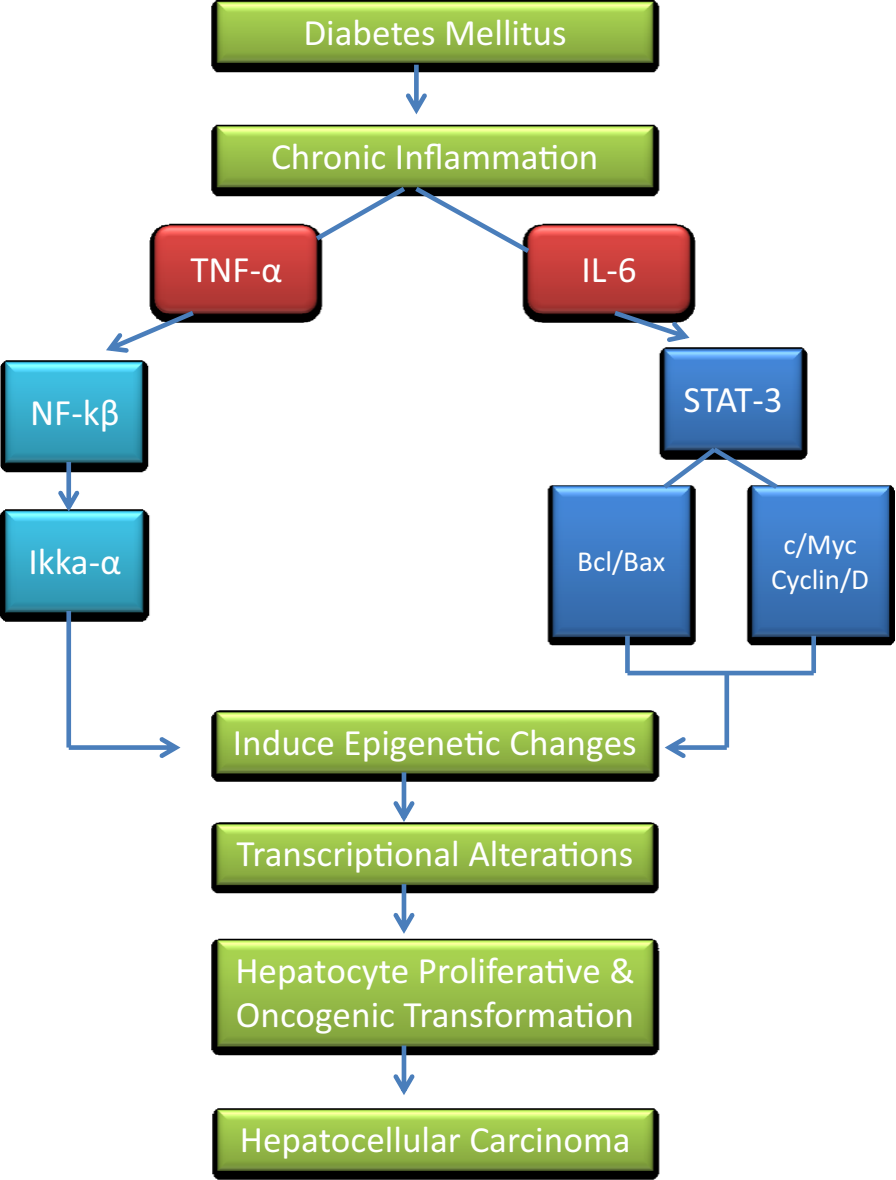
Overview of the possible intermediary mechanisms associated with onset and progression of hepatocellular carcinoma confounded by diabetes mellitus; (i) possible effects of IL-6 and TNF-α as apoptotic and inflammatory markers for the development of HCC and (ii) role of epigenomic reprogramming that drives hepatocellular transformation.

In this mini-review, we summarized the intermediary mechanisms conveying the association between DM and HCC development. Whereby, in the first section, we highlighted that DM can work as a promoter for HCC development since number of evidence lines showed that it may worsen the HCC progression as evident by co-existence of pro-inflammatory gene products (TNF-α and IL-6) during hepatocarcinogenesis. These inflammatory markers play a role in monitoring levels of apoptotic regulators (Bcl2 and Bax), suggesting their importance as apoptotic and inflammatory markers for HCC. The second section, we have focused on the possible effects of epigenetic mechanisms of HCC upon co-existence of DM. Number of studies showed that DNA methylation, histone modifications, and RNA interference, may lead to activation of pro-inflammatory signaling (NF-κB and JAK/STAT) and deregulation of metabolic pathways. The crosstalk of chromatin-modifying enzymes, microRNAs, signaling pathways and the downstream transcription factors may result in epigenomic reprogramming that drives hepatocellular transformation. Many of the epigenetic events and methylated genes described previously in other studies have been detected in premalignant tissues and in the serum of patients prior to or concurrent with the diagnosis of cancer. For this reason, DNA methylation, hypomethylation or histone modifications are attractive candidates for early-detection biomarkers (Figure 2).

## Future trends

Inflammatory markers (TNF-α and IL-6) can be suggested to be used as diagnostic tools for HCC along with alpha-fetoprotein (AFP) that may help to monitor the severity of hepatocellular damage and hepatic function in diabetic patients. These markers can also help to follow-up patients diagnosed with HCC prognostically. On the other hand, epigenetic alterations could be used as biomarkers of early-detection for HCC which may help in prognosticate diabetic patient outcomes or even as novel therapeutic compounds. However, large-scale prospective data is warranted before this approach can be translated into personalized medicine.

## Competing interests

All authors have nothing to disclose.

## Author’s contribution

MMAK was involved with literature review to interpret the association, drafting of the manuscript and critical revision of the manuscript. RA, OA and KB were involved with drafting of the manuscript and critical revision of the manuscript. All authors read and approved the final manuscript.
